# Finishing Proximal Cement Fillings

**Published:** 1898-11

**Authors:** 


### Finishing Proximal Cement Fillings.

Pass a thin piece of celluloid, oiled, between the teeth, and after introducing the cement into the cavity, press the celluloid firmly over the cavity when the cement is fairly stiff, and hold in this position for a few moments. The oil prevents the cement sticking to the matrix, and the matrix gives the proper space between the teeth.—*British Journal of Dental Science.*

